# Gastric metastasis of malignant melanoma with osteosarcomatous differentiation: a rare and unusual presentation

**DOI:** 10.1097/CMR.0000000000001094

**Published:** 2026-04-28

**Authors:** Vidya Monappa, Badareesh L., Balaji Musunuri, Naveen Salins

**Affiliations:** a Department of Pathology; b Department of Surgery; c Department of Gastroenterology; d Department of Palliative Medicine, Kasturba Medical College, Manipal Academy of Higher Education, Manipal, Karnataka, India

**Keywords:** gastric metastasis, melanoma, osteosarcomatous differentiation, SATB2

## Abstract

Gastric metastasis of melanoma is a rare event associated with poor prognosis and short survival. Patients usually present with gastrointestinal symptoms, and in the absence of a prior history of primary tumor elsewhere, this can pose a diagnostic challenge. Melanoma has an inherent property to show transdifferentiation, which is retained even at the site of metastasis. Osteosarcomatous differentiation in melanoma is extremely uncommon, restricted to isolated case reports in the literature. A 79-year-old male patient, a known case of gastric mucosa-associated lymphoid tissue lymphoma, presented with generalized weakness and dysphagia of 15 days duration. Clinical impression was of lymphoma progression. Upper gastrointestinal endoscopy showed a 4 × 5 cm proliferative growth in the gastric fundus. Biopsy of the lesion was suggestive of melanoma with osteosarcomatous differentiation. It was regarded as metastasis as patient had a history of acral lentiginous melanoma 2 years ago. Patient was, however, willing only for palliative treatment. Patient succumbed to septic shock within 2 months of diagnosis. Herein, we report a case of gastric metastasis of melanoma with osteosarcomatous differentiation and discuss the clinical presentation, radiologic findings, challenges in histopathological diagnosis, and treatment options for this unusual entity.

## Introduction

Gastric metastasis is a rare event with a poor prognosis and a reported incidence of less than 1% [1]. The gastrointestinal tract is an uncommon site for metastasis, most frequently from adenocarcinomas of the breast, lung, liver, and kidney, and from malignant melanoma. Melanoma metastasis in the gastrointestinal tract commonly involves the small intestine and colon, whereas gastric metastasis is extremely rare, with less than 120 cases reported in literature [2]. Melanoma is well recognized for its capacity for divergent morphological differentiation, including Schwannian, fibroblastic, smooth muscle, rhabdomyoblastic, and osteocartilaginous lineages, thereby significantly complicating histopathological diagnosis.

## Case report

A 79-year-old male patient, a known case of gastric mucosa-associated lymphoid tissue lymphoma, presented with dysphagia and generalized weakness with loss of appetite and weight loss for 15 days. Laboratory investigations revealed hemoglobin of 6.9 g/dl, hypoalbuminemia, and elevated total white blood cell counts. He had a history of acral lentiginous melanoma of the right foot, for which he underwent ray amputation of third, fourth, and fifth toe of right foot with popliteal lymph node dissection 2 years ago. The tumor was pT4a, N0, Clarks level IV. Patient did not receive further additional treatment.

**Fig. 1:**
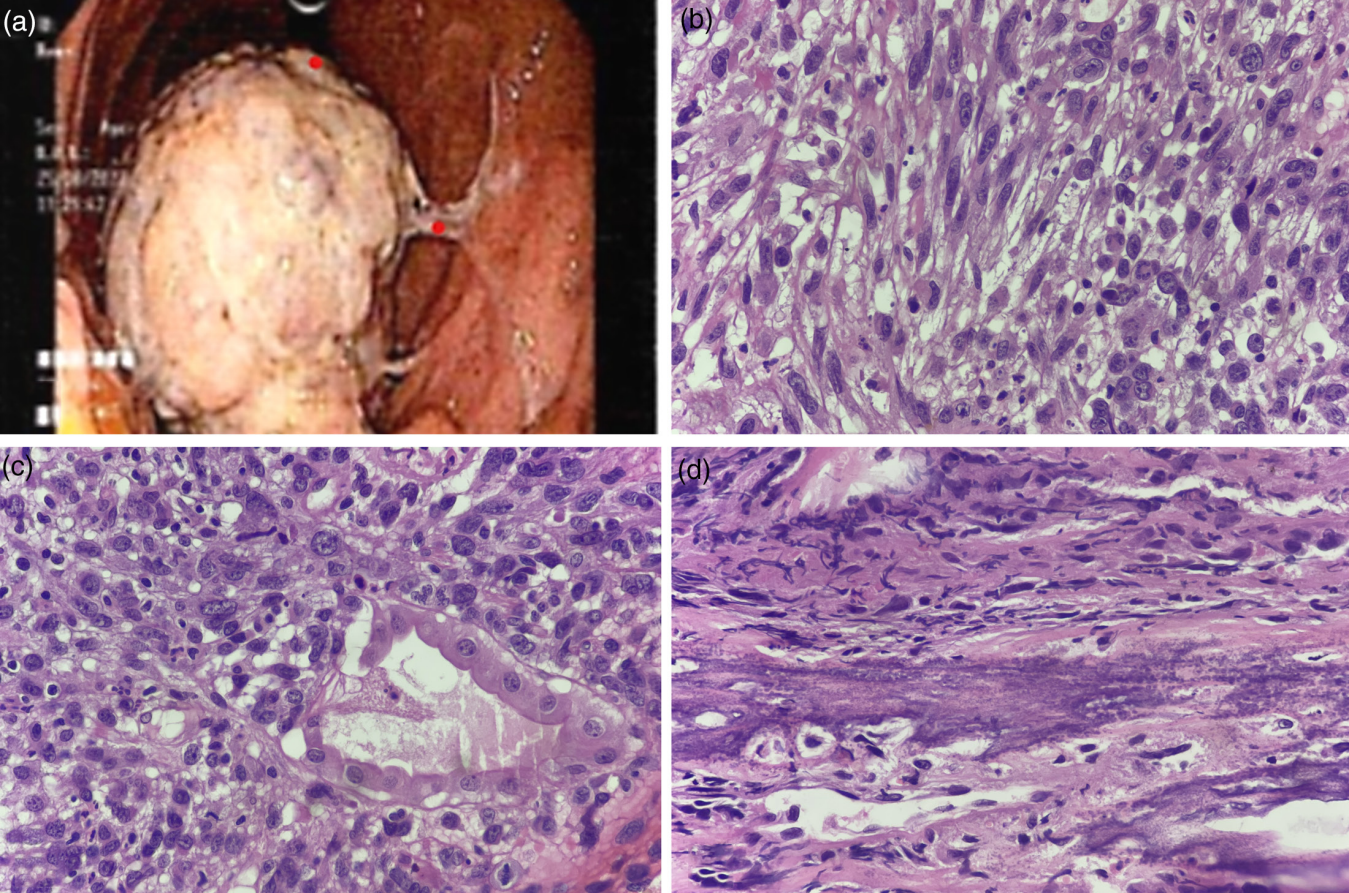
(a) Endoscopy image showing friable fleshy proliferative growth measuring 4 × 5 cm, covered with slough in the fundus. (b) Section showing fascicles of pleomorphic spindle cells; H&E, 400×. (c) Section showing plump epithelioid cells with coarse chromatin, prominent nucleolus and moderate cytoplasm diffusely infiltrating between gastric glands; H&E, 400×. (d) Section showing malignant osteoid and pleomorphic cells. H&E; 400×. H&E, hematoxylin and eosin.

Upper gastrointestinal endoscopy revealed curdy white precipitates in the esophagus suggestive of candidiasis along with a large, friable, fleshy, multilobulated proliferative growth measuring 4 × 5 cm, covered with slough noted in the fundus along the greater curvature (Fig. 1a). Biopsy was performed, and the sample was sent for histopathological examination.

### Histopathology findings

The sections revealed a tumor composed of sheets of pleomorphic epithelioid to spindle cells with enlarged pleomorphic nuclei and granular chromatin, with prominent nucleoli, few binucleate and multinucleated forms, and abundant amphophilic to clear cytoplasm with diffuse infiltration between the glands. Focal malignant osteoid was noted (Fig. 1b–d). Extensive areas of necrosis, increased and atypical mitosis were observed.

**Fig. 2:**
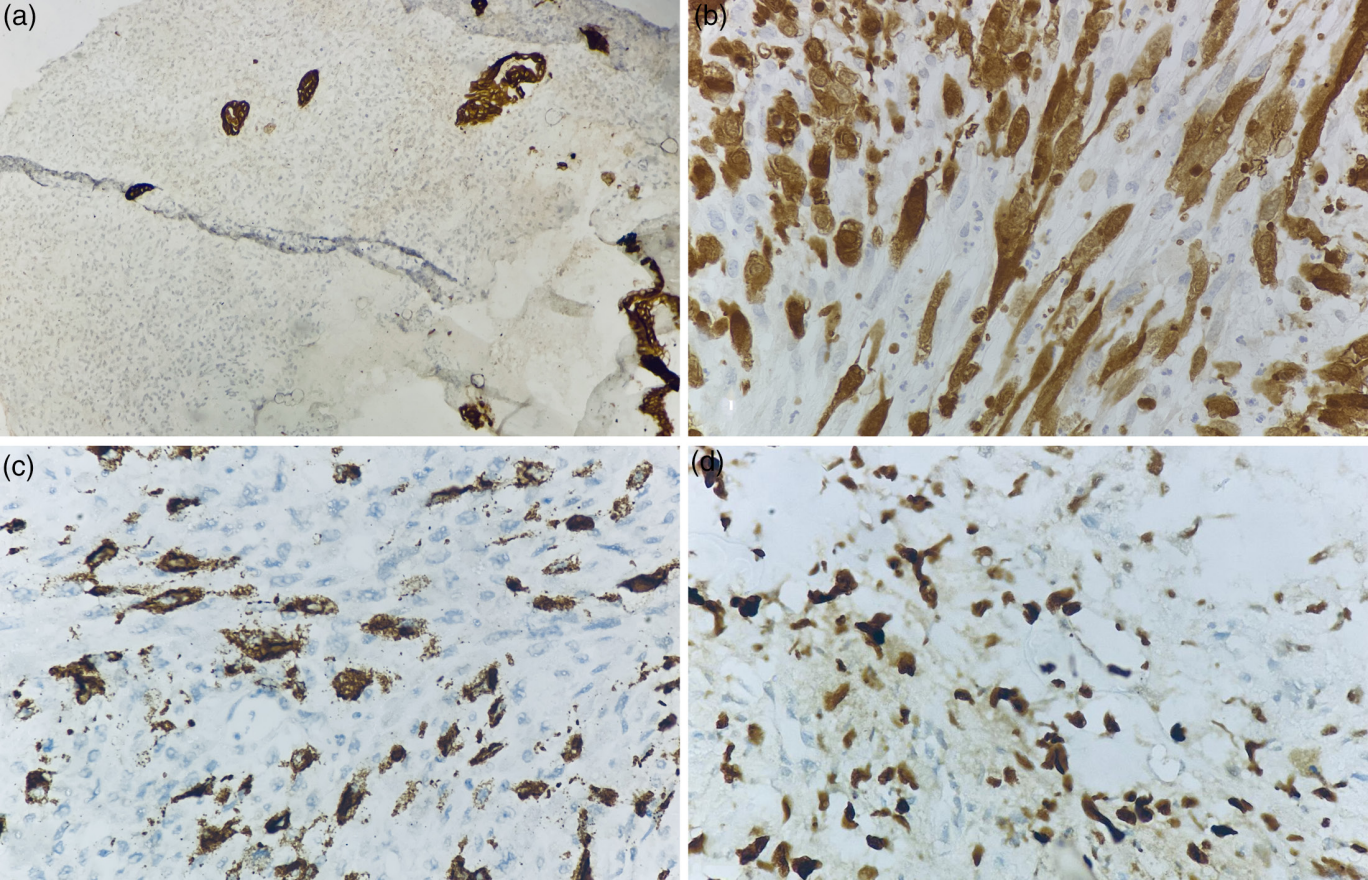
Immunohistochemistry images showing that tumor cells are (a) CK negative; 200×; (b) S100 nuclear and cytoplasmic positive; 400×; (c) HMB45 cytoplasmic positive; 400×; and (d) SATB2 nuclear positive; 400×.

The tumor cells were immunopositive for HMB45 and S100 and focally positive for C-KIT while being negative for CK, LCA, and *BRAF*. SATB2 showed nuclear positivity in cells around malignant osteoid (Fig. 2). A diagnosis of metastatic melanoma with osteosarcomatous differentiation was made.

PET computed tomography revealed multiple hypermetabolic lesions in the right external iliac lymph node, stomach, distal end of the pancreas, gastrohepatic ligament, presplenic ligament, and bilateral lungs suggesting widespread metastatic disease.

Treatment received: the patient party was counselled and explained the patient’s prognosis. The patient refused immunotherapy and wanted to proceed with only palliative care.

One month later, the patient presented with shortness of breath with cough and was admitted for supportive management at the Department of Palliative Medicine and Supportive Care. He was diagnosed with aspiration pneumonia, acute kidney injury, disseminated intravascular coagulopathy, and progressed to septic shock despite extensive intervention. He died within 5 days of admission.

## Discussion

Metastatic tumors involving the stomach are rare and pose significant diagnostic challenges both clinically and histologically. Gastric metastasis may present with gastrointestinal/systemic symptoms. Digestive tract bleeding (hematemesis, melena, iron deficiency anemia, and weight loss), however, occur more frequently [3]. The overall reported incidence of gastric metastasis is 0.2–0.7% [1,4]. Frequently described primaries include adenocarcinomas of breast, lung, liver, renal, and melanoma [4,5]. Gastrointestinal tract metastases of melanoma are a poor prognostic factor and have been described in approximately 60% of autopsies [6,7]. Gastric metastasis, however, is extremely rare and is mostly restricted to autopsy cases, with very few isolated reports/series in the literature describing the clinical and endoscopy findings. The present case is one such rare example, describing clinical, endoscopy, and pathological findings.

Reggiani *et al*. [2] in their systematic review on the gastric metastasis in melanoma patients (*N* = 113) reported that the skin, eyes, and mucous membranes were the most common sites of primary melanoma. The 2-year survival rate in these patients was only 4%, with a median survival duration of only 3 months. Sun and Liu [5] reported that melanoma involving the skin of hands, feet, and eyes was the most common primary site in patients with gastric metastasis. The present patient had a history of acral lentiginous melanoma and died within a period of 2 years since the primary diagnosis. Most of these patients also had other system involvement, which was also observed in the present case. Gastric body involvement is more common than antral metastasis (5%) [2]. In our case also, gastric fundic lesion was noted.

Endoscopy plays a useful diagnostic role. Characteristic findings include pigment and the ‘bull’ eye sign. Many newer classifications have been proposed by Nelson and Lanza [8] and Oda *et al*. [9] (1 – polypoid, 2 – ulcerated, well-defined margins, 3 – ulcerated with ill-defined margins, and 4 – diffuse). The present case was a polypoidal lesion. Unlike primary adenocarcinomas, metastatic tumors usually present as mucosal edema/thickening and submucosal nodularity [10].

Histopathologic examination may show cells with cytoplasmic dusty brown/black melanin pigment. Melanoma is known to undergo divergent differentiation – including Schwannian, fibroblastic, smooth muscle, and rhabdomyoblastic lineages. Osteocartilaginous differentiation in melanoma was first noted 35 years ago. Since then, less than 30 cases have been reported. Gallagher *et al*. [11] described eight cases of melanoma with osteocartilaginous differentiation (six primary and two metastatic) and reported that staining with the osteoblastic marker SATB2 ranged from negative to diffuse positive. However, no distinct molecular alterations were noted in these cases. Our case showed focal positivity for SATB2. The exact mechanism of this differentiation is still unclear. One hypothesis is that it is a reactive process to inflammation or trauma at the site. However, SATB2 positivity is associated with the acquisition of an osteoblastic differentiation program by tumor cells.

Currently, gastric metastasis of melanoma patients are treated with local surgical resection and chemotherapy. Recently, the use of targeted therapies, such as BRAF inhibitors and immune checkpoint inhibitors, has increased the survival of patients with metastatic melanoma [3].

In conclusion, gastric metastasis is a rare event associated with poor prognosis and survival in patients with melanoma. It is commonly associated with primary cutaneous or uveal melanoma. Like melanoma elsewhere, gastric metastasis can have diverse morphologies, including osteogenic differentiation. This case emphasizes the importance of careful documentation of a patient’s oncologic history, as knowledge of a prior acral melanoma was pivotal in arriving at an accurate and timely diagnosis.

## Acknowledgements

V.M., B.L., and B.M.: conceptualized, diagnosis and treatment. V.M. and B.L.: manuscript writing and data collection. All authors reviewed the manuscript.

As this is a single case report, the institutional ethics committee approval was waived off.

Consent for publication was taken from all stakeholders.

### Conflicts of interest

There are no conflicts of interest.
